# The effects of exercise on hypothalamic neurodegeneration of Alzheimer’s disease mouse model

**DOI:** 10.1371/journal.pone.0190205

**Published:** 2018-01-02

**Authors:** Khoa Do, Brenton Thomas Laing, Taylor Landry, Wyatt Bunner, Naderi Mersaud, Tomoko Matsubara, Peixin Li, Yuan Yuan, Qun Lu, Hu Huang

**Affiliations:** 1 Department of Kinesiology, East Carolina University, Greenville, North Carolina, United States of America; 2 East Carolina Diabetes and Obesity Institute, East Carolina University, Greenville, North Carolina, United States of America; 3 Department of Anatomy and Cell Biology, Brody School of Medicine, East Carolina University, Greenville, North Carolina, United States of America; 4 The Harriet and John Wooten Laboratory for Alzheimer’s and Neurodegenerative Diseases Research, Brody School of Medicine, East Carolina University, Greenville, North Carolina, United States of America; 5 Department of Physiology, Brody School of Medicine, East Carolina University, Greenville, North Carolina, United States of America; Universidad Pablo de Olavide, SPAIN

## Abstract

Alzheimer’s disease is a neurodegenerative disorder that affects the central nervous system. In this study, we characterized and examined the early metabolic changes in the triple transgenic mouse AD model (3xtg-AD), and their relationship with the hypothalamus, a key regulator of metabolism in the central nervous system. We observed that the 3xtg-AD model exhibited significantly higher oxygen consumption as well as food intake before reported amyloid plaque formation, indicating that metabolic abnormalities occurred at early onset in the 3xtg-AD model compared with their counterparts. Analysis of gene expression in the hypothalamus indicated increased mRNA expression of inflammation- and apoptosis-related genes, as well as decreased gene expression of Agouti-related protein (AgRP) and Melanocortin 4 receptor (MC4R) at 12 weeks of age. Immunofluorescence analysis revealed that pro-opiomelanocortin (POMC) and NPY-expressing neurons decreased at 24 weeks in the 3xtg-AD model. Four weeks of voluntary exercise were sufficient to reverse the gene expression of inflammation and apoptotic markers in the hypothalamus, six weeks of exercise improved glucose metabolism, moreover, 8 weeks of voluntary exercise training attenuated apoptosis and augmented POMC and NPY-expressing neuronal populations in the hypothalamus compared to the control group. Our results indicated that early onset of metabolic abnormalities may contribute to the pathology of AD, which is associated with increased inflammation as well as decreased neuronal population and key neuropeptides in the hypothalamus. Furthermore, early intervention by voluntary exercise normalized hypothalamic inflammation and neurodegeneration as well as glucose metabolism in the 3xtg-AD model. The data, taken as a whole, suggests a hypothalamic-mediated mechanism where exercise prevents the progression of dementia and of Alzheimer’s disease.

## Introduction

Alzheimer’s disease (AD) is a debilitative neurological disease that is strongly associated with aging [1]. It is characterized by neurodegeneration associated with amyloid beta plaque formation and neurofibrillary tangles (NFTs) derived from tau phosphorylation in the CNS. It is still under debate whether hyper-phosphorylation of tau and the buildup of proteins, like amyloid beta, are the main causes of AD. AD symptoms not directly related to the buildup of detrimental protein have not been fully explored yet. For instance, alterations in circadian rhythm and mitochondrial function have been observed during the onset of Alzheimer’s [2].

Metabolic etiologies have always been a focus in the study of AD. It has been reported that a high-fat diet induces metabolic dysfunction in the AD model leading to aggravation in the pathology of amyloid beta aggregation and cognitive dysfunction [3]. On a larger scale, AD shares many similar characteristics to metabolic diseases, such as type 2 diabetes mellitus (T2DM). Some of these characteristics include inflammation, oxidative stress, and altered insulin signaling [4]. In T2DM, obesity and high-fat diets lead to peripheral inflammation, which adds to cellular stress leading to insulin resistance. In AD, amyloid beta leads to microglia and astrocyte activation, resulting in inflammation and insulin resistance in the CNS. This contributes to synapse deterioration and cognitive decline, also seen in T2DM [4]. On the other end of the metabolic spectrum, such as caloric restriction (CR), has been shown to have a beneficial role in retarding aging-related diseases like AD, most likely via enhancing metabolic function throughout the body, including but not limited to, reduced abdominal fat mass, improved insulin sensitivity, and reduced levels of pro-inflammatory cytokines [5]. Researchers have also sought to combine the beneficial responses of exercise with AD for a long time to counteract the negative effects of AD. Many different studies have pursued exercise therapy to slow down or reverse AD, with varying degrees of success [6, 7]; however, the detailed mechanisms of the effects of exercise on AD are still not fully understood.

To date, there has been relatively little study into the metabolic effects of AD in the early stages. Obesity in midlife increases the risk of developing AD and dementia by nearly 100% in human subjects-based meta-analysis [8]. In rodents, early mitochondrial dysfunction occurs, preceding the normal pathology in AD models [2]. It has been observed that a sudden infusion of amyloid beta directly resulted in a decrease in mitochondrial membrane viscosity, associated decrease in ATP, and inhibition of the respiratory chain in neuronal cell cultures [9]. These studies indicated that the metabolic alteration at early age may lead to the progression of dementia and AD.

It is believed that the pathology of AD usually occurs earliest in the cortex and hippocampus, areas responsible for spatial coordination and memory, and slowly spreads throughout the central nervous system (CNS). However, little is known about the neuronal alterations in the hypothalamus, a key area of metabolic regulation. Two important populations of neurons in the hypothalamus that regulate metabolic homeostasis are POMC and AgRP/NPY-expressing neurons. POMC-expressing neurons release several different neuropeptides including α-Melanocyte-stimulating hormone (α-MSH), which is responsible for suppressing appetite through the promotion of a “satiety” response in the paraventricular nucleus (PVN) via melanocortin 4 receptors (MC4R) signaling pathway [10]. AgRP/NPY neurons release neuropeptide Y, Agouti-related peptides, as well as GABA to promote appetite and to decrease energy expenditure [11].

It is well regarded that metabolic dysfunction plays a role in AD. It has been shown in different mice models that metabolic deficiencies precede the pathology of AD. In the present study, using the 3xtg-AD mouse model, we pinpointed a time-period when significant metabolic discrepancies occurred. We further demonstrated that these effects are associated with the changes in the hypothalamic neuronal populations that are involved in metabolic regulation. We also sought to investigate the effects of voluntary exercise on hypothalamic neurons that are related to the early metabolic alterations in the 3xtg-AD mouse model.

## Materials and methods

### Experimental animals

The triple transgenic AD mice models that display three mutations associated with AD (APP Swedish, MAPT P301L, and PSEN1 M146V) and their control mice (129/C57BL6) were provided by The Harriet and John Wooten Laboratory for Alzheimer's and Neurodegenerative Diseases Research at East Carolina University [12]. All animals were originally from The Jackson Laboratory (Bar Harbor, ME, USA) [13] and were confirmed for their genotype by PCR. They were housed under controlled temperature and lighting conditions of 20–22 degrees and 12-h light-dark cycle. For the study of metabolic status, 12 TGX3 male mice and 10 control mice were used at 10, 12 and 24 weeks of age. For the 4 weeks of voluntary wheel running study, 6 control male mice and 14 TGX3 mice were used at 8 weeks of age. The 14 TGX3 male mice were further divided into 2 groups, a sedentary group (N = 7) and a 4 weeks’ exercise training program (N = 7). For an 8 week of voluntary wheel running study, another set of 6 control male mice and 14 TGX3 mice were used at 16 weeks of age. The 14 TGX3 male mice were further divided into 2 groups, a sedentary group (N = 7) and an 8 weeks’ exercise training program (N = 7). The age-matched male mice in the AD + Ex group were placed in cages equipped with running wheels for mice (TSE PhenoMaster, TSE Systems, Homburg vor der Höhe, Germany), whereas mice in the control group and AD sedentary group were housed in cages without running wheels for 4 and 8 weeks, respectively. Each cage accommodated one mouse. All aspects of animal care and experimentation were conducted in accordance with the National Institutes of Health Guide for the Care and Use of Laboratory Animals and approved by the Institutional Animal Care and Use Committees of East Carolina University.

### Metabolic measurements

Food intake was measured over a 14-day period. Eight mice per group were given ~10–12 g of food every day and cages were changed every time when the food weight was measured. Any residual bits of food in the bedding were not included in the measurements. The data were combined, averaged, and analyzed, and cumulative food intake data were obtained by adding all intake measurements during the study. Fat and lean body mass were assessed using Echo MRI (Echo Medical Systems Houston, USA). Energy expenditure was measured by assessing oxygen consumption and carbon dioxide production with indirect calorimetry by using CLAMS (TSE PhenoMaster, TSE Systems, Homburg vor der Höhe, Germany). The mice were acclimated in the CLAMS chambers for 72 h before data collection, and had free access to food and water for the duration of the study.

### Glucose tolerance test and glucose level measurement

Two weeks prior to the last day of the 8 weeks’ exercise training experiment, an intraperitoneal glucose tolerance test (IPGTT) was performed. After an overnight fast, IPGTT was performed by intraperitoneal injection of a 20% glucose solution (1g/kg). Tail blood samples were collected before and 15, 30, 60, 90, and 120 minutes after the injection. After a week recovery, a fed status glucose level and an over-night fasted glucose level were measured by tail blood samples at 8:00–8:30 am at 15 weeks of age. A glucose meter was used to measure blood glucose levels (Relion prime Blood Glucose Monitoring System, ARKRAY INC. Kyoto, JAPAN).

### Tissue collection

Three mice from each group in the exercise training study were euthanized by ketamine/xylazine and perfused intracardially with phosphate-buffered saline (PBS), followed by 10% formalin by the end of experiments. The brains were then collected and fixed in 10% formalin overnight, and finally transferred in 30% sucrose solution. For the gene expression analysis, three mice’s whole hypothalamuses per each group in the exercise training study were isolated and removed by the end of experiments, and submerged in liquid nitrogen to be preserved for qPCR later.

### Immunofluorescence staining

For fluorescence detection of amyloid-beta, NeuN, POMC, and NPY, 30-um coronal brain sections in three groups were prepared and immunohistochemistry was performed as previously described (14). Briefly, brain sections were incubated with antibody to NeuN (Cell Signaling, Danvers, MA, USA), POMC (Phoenix Pharmaceuticals, Burlingame, CA, USA), NPY (Santa Cruz, Dallas, TX USA) and further incubated with fluorescent-labeled secondary antibodies. Stained sections were photographed digitally using an optical microscope (Leica DM6000, Wetzlar, Germany), and the images were transferred to a computer medium. NeuN, POMC, and NPY-positive neurons throughout the medio-basal hypothalamus were counted. Quantification of immunofluorescent images was obtained using ImageJ Cell Counter plug-in function for marking and numbering positive cells. For each brain, at least three sections containing arcuate nucleus were double-blind analyzed. using the ImageJ software.

### Quantitative PCR

The expression of specific hypothalamic mRNAs was analyzed using quantitative real-time PCR (RT-qPCR) (POWER-SYBR GREEN PCR Master Mix; Applied Biosystems, Foster City, CA, USA) as previously described [14]. Quantification reactions were performed in duplicate for each sample using the ‘delta-delta Ct’ method. Glyceraldehyde 3-phosphate dehydrogenase (GAPDH) was chosen as a reference (housekeeping) gene. Primers used for QPCR are listed in S1 Table.

### TUNEL assay

A terminal deoxynucleotidyl-transferase-mediated dUTP nick end-labeling (TUNEL) assay was used to identify double-stranded DNA fragmentation. Briefly, after being washed in PBS, samples were incubated at 4 degrees in permeability solution (PBS, 0.1% triton-x, 0.1% sodium citrate) for two minutes, and then incubated with tunnel assay solution (In Situ Cell Death Detection Kit, Fluorescein, Sigma-Aldrich, St. Louis, MO, USA) for one hour at 37 degrees. After being washed in PBS, all sections were mounted on slides with vectashield anti-fade reagent. Negative and positive control for the TUNEL assay were confirmed by staining the sections in the same manner without primary antibody (negative control) or pretreated with DNAse I (positive control). Counts were done by creating a square section of the same size on each image. A TUNEL positive cell was denoted in the ARC from slides (n = 3) of each group.

### Statistical analysis

The graphs represent means ± SEM, and significance was determined through non-paired t-test, after one-way ANOVA, as applicable. Significance was set at P ≤ .05.

## Results

### 3Xtg-AD mice exhibit amyloid pathology and arcuate nucleus neurodegeneration

**Fig 1:**
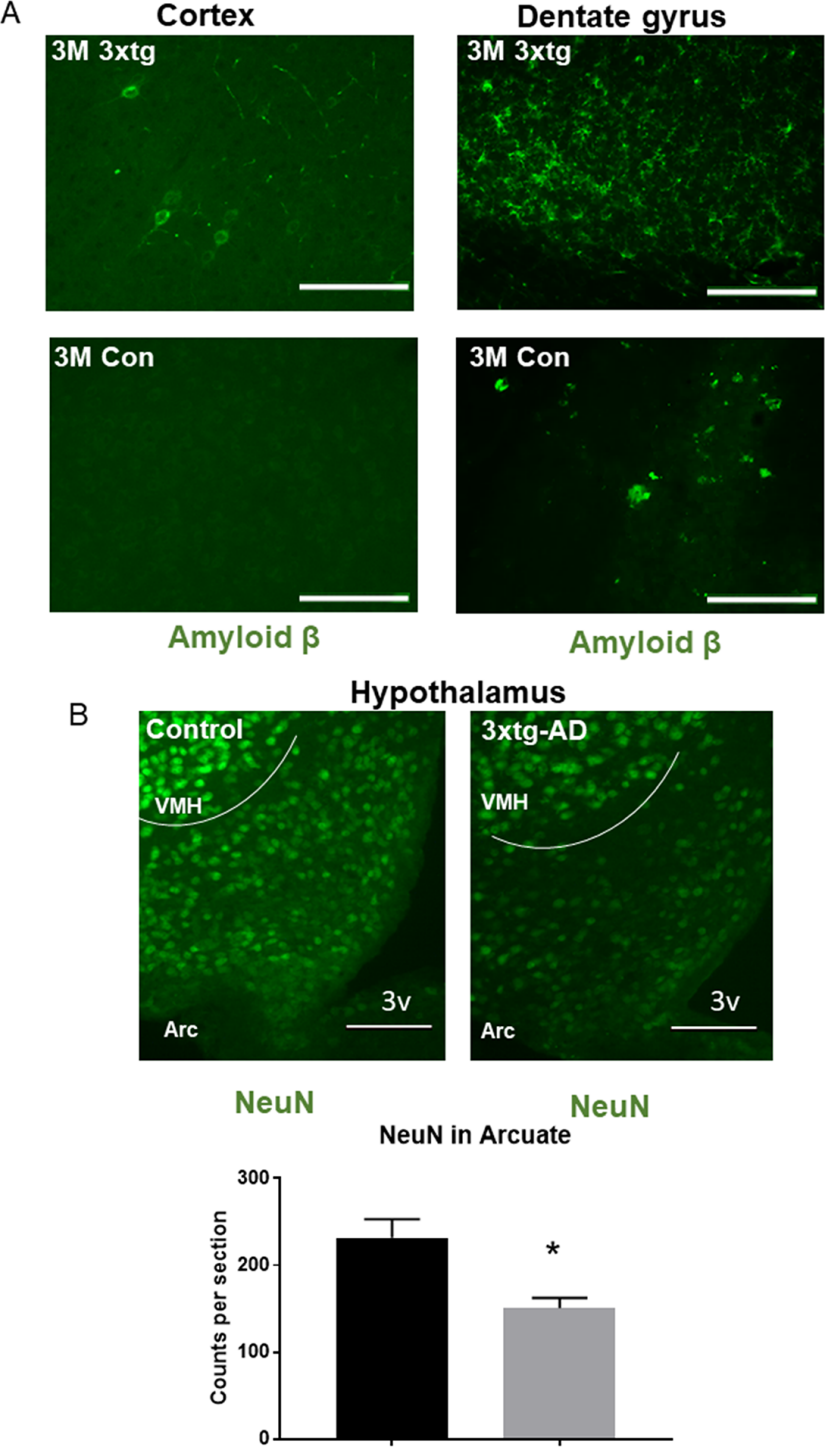
A comparison of amyloid beta in cortex and dentate gyrus area of hippocampus and NeuN positive cell in arcuate nucleus between the triple transgenic (3xtg-AD) and control. A. Mice with the 3xtg-AD genes (top) showed amyloid beta under fluorescent microscopy compared to control (bottom). Representative image of cortex (left) and dentate gyrus area of hippocampus(Right) showed amyloid beta congregation around neurons in the 3xtg-AD and few in the control. B. Representation of arcuate nucleus neuron population (NeuN positive) from the control, 3xtg-AD at 20 weeks of age. Arc: Arcuate nucleus of hypothalamus; VMH: Ventromedial nucleus of the hypothalamus; 3V: third ventricle, scale bars represent 200 μM.

Amyloid pathology is one of the earliest indicators of AD. Amyloid beta, through several mechanisms, is overexpressed in the form of oligomers. These oligomers interfere with synaptic transmission and form amyloid plaques. In the 3xtg-AD model, we observed amyloid beta pathology in the cortex and dentate gyrus area of hippocampus as early as 12 weeks compared with the control mice, but no amyloid plaques were detected (Fig 1A). Any amyloid beta pathology was not detected in the hypothalamic area of 3xtg-AD (data not shown). In the arcuate nucleus of hypothalamus, we observed arcuate NeuN positive cells were remarkably reduced in the 3xtg-AD mice at 20 weeks of age compared with the control mice (control: 232 ± 12 versus 3xtg AD: 151 ± 7; Fig 1B).

### Changes in energy metabolism in 3Xtg-AD Mice

**Fig 2:**
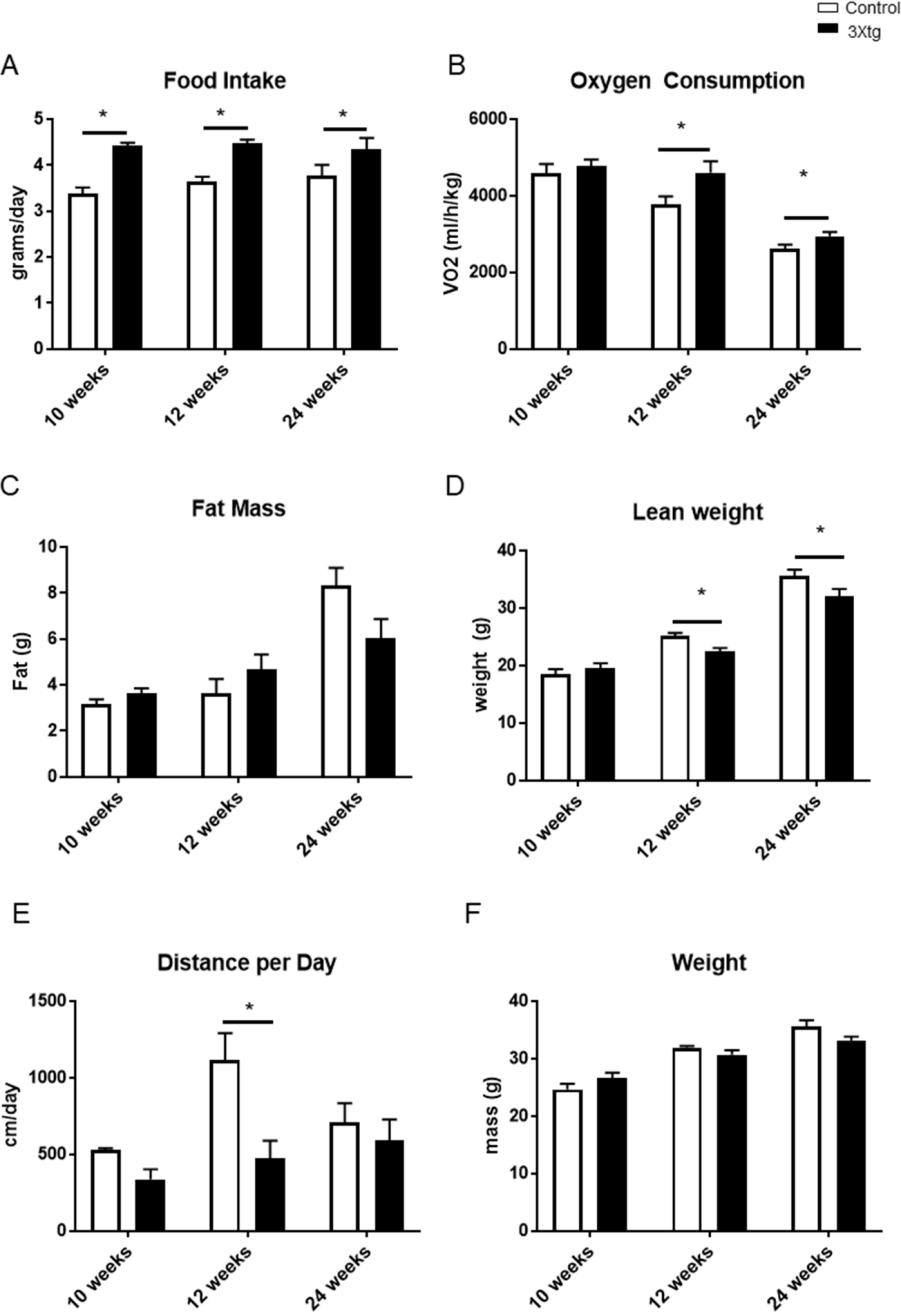
Metabolic status at different age points (10, 12, and 24 weeks) in the triple transgenic (3xtg-AD) and control. 2A. Food intake, 2B. Oxygen consumption, 2C. Fat mass, 2D. Lean mass, 2E. Running distance per day, 2F. Body weight at 10, 12, and 24 weeks between groups. N = 4–6 per group, *p< 0.05 vs Control. Data represent mean ± SEM.

At different time points, the 3xtg-AD mice model consumed significantly more food than their counterparts (10 weeks; control: 3.4 g ± 0.1 versus 3xtg AD: 4.4 g ± 0.1; 12 weeks; control: 3.6 g ± 0.1 versus 3xtg AD: 4.5g ± 0.1; 24 weeks; control 3.8 g ± 0.2 versus 3xtg AD: 4.3g ± 0.2) (Fig 2A). Oxygen consumption measurements provided insight to the metabolic changes in the 3xtg-AD model starting from 12 weeks of age compared to controls; differences persist at 24 weeks of age (10 weeks; control: 4590 ml/h/kg ± 245 versus 3xtg AD: 4768 ml/h/kg ± 180; 12 weeks; control: 3770 ml/h/kg ± 217 versus 3xtg AD: 4601 ml/h/kg ± 303; 24 weeks; control: 2630 ml/h/kg ± 97 versus 3xtg AD: 2947 ml/h/kg ± 104) (Fig 2B). Increased oxygen consumption indicated that the 3xtg-AD mice have a higher metabolic rate than their counterparts. Locomotion activity data showed a decrease at 12 weeks in the 3xtg-AD model (Fig 2E). Taken together, this demonstrates that the 3xtg-AD mice consumed and expended more energy than their control counterpart, indicating that there were metabolic abnormalities occurring at 12 weeks of age.

There was no difference in total weight (10 weeks; control: 24.7 g ± 1.0 versus 3xtg AD: 26.6 g ± 1.0; 12 weeks; control: 31.9 g ± 0.3 versus 3xtg AD: 30.7 g ± 0.8; 24 weeks; control: 35.6 g ± 1.0 versus 3xtg AD: 33.2 g ± 0.7) (Fig 2F) and fat mass (10 weeks; control: 3.1 g ± 0.2 versus 3xtg AD: 3.6 g ± 0.2; 12 weeks; control: 3.6 g ± 0.6 versus 3xtg AD: 4.7 g ±0.7; 24 weeks; control: 8.3 g ± 0.8 versus 3xtg AD: 6.0 g ± 0.9) between both groups (2C). Lean mass was significantly lower in the 3xtg-AD mice at 12 and 24 weeks of age (10 weeks; control: 18.5 g ± 0.9 versus 3xtg AD: 19.5 g ± 0.8; 12 weeks; control: 25.1 g ± 0.6 versus 3xtg AD: 22.4 g ± 0.7; 24 weeks; control: 35.7 g ± 1.0 versus 3xtg AD: 32.0 g ± 1.3) (Fig 2D).

### Gene expressions in the hypothalamus of 3Xtg-AD Mice

**Fig 3:**
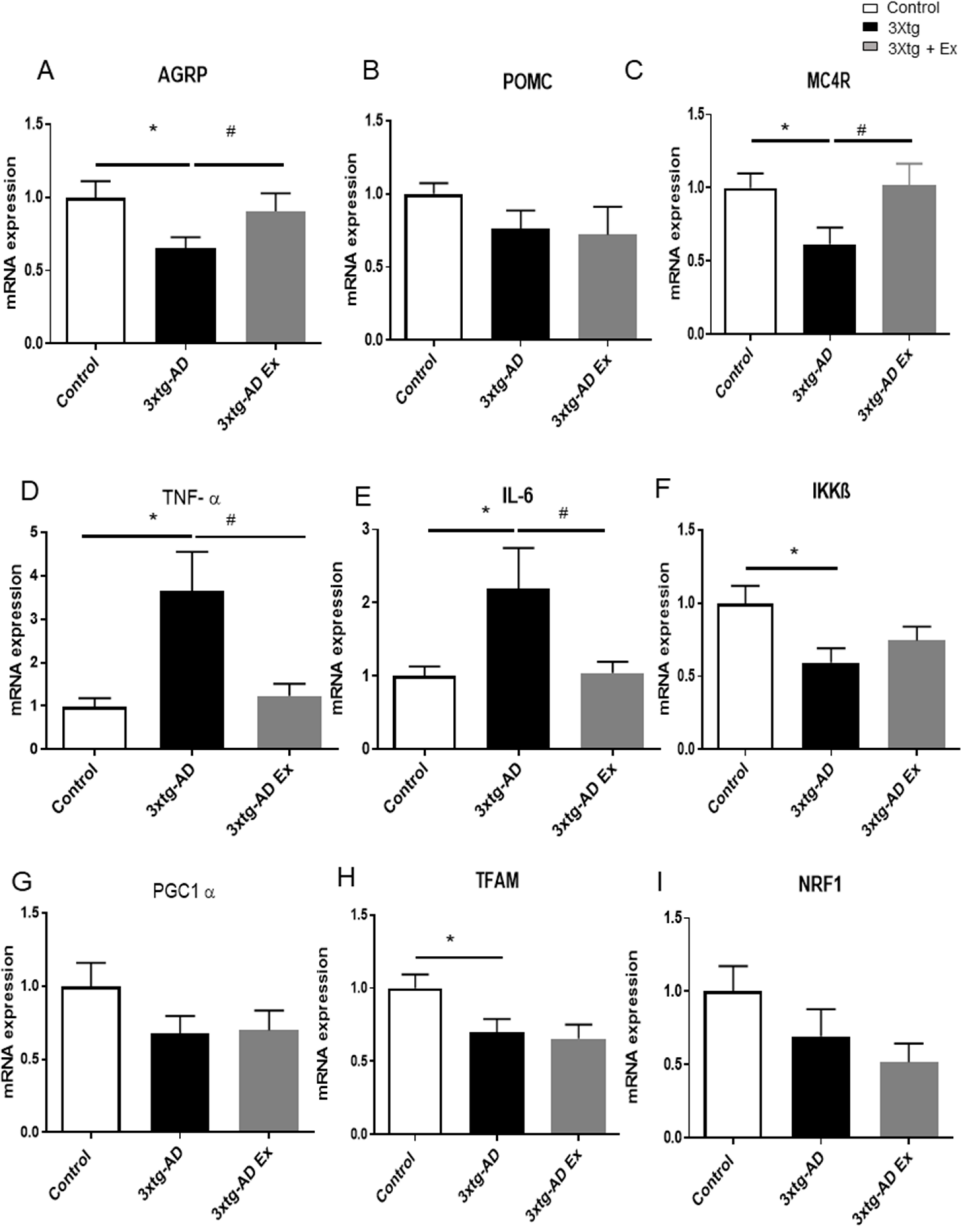
Gene expression in the hypothalamus at 12 weeks after a 4-week voluntary exercise treatment in the triple transgenic (3xtg-AD) and control. 3A. mRNA expression of AgRP, 3B. POMC, 3C MC4R, 3D. TNF-α, 3E. IL-6, 3F. IKKβ, 3G. PGC1 α, 3H. TFAM, 3J. NRF1 at 12 weeks among control, 3xtg-AD, and a 3xtg-AD group given a voluntary exercise treatment. N = 4–6 per group, *p< 0.05 vs Control, #p < 0.05 vs 3xtg-AD group. Data represent mean ± SEM.

At 12 weeks, the 3xtg-AD mice displayed decreased AgRP and MC4R mRNA expression (AgRP: control: 1.0 ± 0.1 versus 3xtg AD: 0.6 ± 0.1; MC4R: control: 1.0 ± 0.1 versus 3xtg AD: 0.6 ± 1.1) (Fig 3A and 3C). There was no significant difference in the mRNA expression of POMC (control: 1.0 ± 0.1 versus 3xtg AD: 0.8 ± 0.1) (Fig 3B). A short 4-week voluntary exercise training was sufficient in normalizing mRNA of AgRP and MC4R expression but not POMC expression (AgRP: 3xtg AD: 0.6 ± 0.1 versus 3xtg AD + Ex: 0.9 ± 0.1; MC4R: 3xtg AD: 0.6 ± 1.1 versus 3xtg AD+Ex: 1.0 ± 0.2; POMC: 3xtg AD: 0.8 ± 0.1 versus 3xtg AD+Ex: 0.7 ± 0.2) (Fig 3A and 3C).

### Hypothalamus-related degenerative markers

At 12 weeks, Tumor Necrosis Factor alpha (TNF-α) mRNA expression was approximately four times greater than control (control: 1.0 ± 0.2 versus 3xtg AD: 3.7 ±0.9) (Fig 3D). Similarly, interleukin-6 (IL 6), a pro-inflammatory cytokine, was approximately 2.2 times higher than the control (control: 1.0 ± 0.1 versus 3xtg AD: 2.2 ± 0.6) (Fig 3E). IKKβ, an inflammatory and apoptotic marker, decreased in the 3xtg-AD mice (Fig 3F). Since IKKβ inhibits inflammation and apoptosis, the decrease in IKKβ is concurrent with increased inflammation and apoptosis biomarkers. The upregulation in mRNA expression of these biomarkers suggest that apoptosis and inflammatory responses were occurring in the hypothalamus as early as 12 weeks when the metabolic differences were occurring concurrently. 4-week voluntary exercise training reduced TNF-α and IL-6 to similar levels as the control (TNF-α: 3xtg AD: 3.7 ± 0.9 versus 3xtg AD + Ex, 1.2 ± 0.3; IL-6: 3xtg AD: 2.2 ± 0.6 versus 3xtg AD+Ex: 1.0 ± 0.2) (Fig 3D and 3E).

### Hypothalamic mitochondrial related markers

At 12 weeks, while not statistically significant, there were trends toward decreases in hypothalamic mitochondrial biogenesis-related gene expression; peroxisome proliferator-activated receptor gamma coactivator 1-alpha (PGC1 α), mitochondrial transcription factor A (TFAM), and nuclear respiratory factor 1 (NRF1) in the 3xtg-AD mice compared with their control (PGC1 α; control: 1.0 ± 0.2 versus 3xtg AD: 0.7 ± 0.1; TFAM; control: 1.0 ± 0.1 versus 3xtg AD: 0.7 ± 0.1; NRF1; control: 1.0 ± 0.2 versus 3xtg AD: 0.7 ± 0.2) (Fig 3G, 3H and 3J). After 4 weeks of voluntary exercise intervention, there were no differences in mitochondrial biogenesis markers; PGC1 α, TFAM, and NRF1 between the 3xtg-AD mice with exercise and 3xtg-AD mice (PGC1 α; 3xtg AD: 0.7 ± 0.1 versus 3xtg AD + Ex: 0.7 ± 0.1; TFAM; 3xtg AD: 0.7 ± 0.1 versus 3xtg AD+Ex: 0.7 ± 0.1; NRF1; 3xtg AD: 0.7 ± 0.2 versus 3xtg AD+Ex: 0.5 ± 0.1) (Fig 3G, 3H and 3J), suggesting that short term exercise did not augment the key gene expression in mitochondrial biogenesis in 3xtg-AD mice compared to control at early time points.

### 6 weeks’ exercise training improves glucose metabolism in 3Xtg-AD Mice

**Fig 4:**
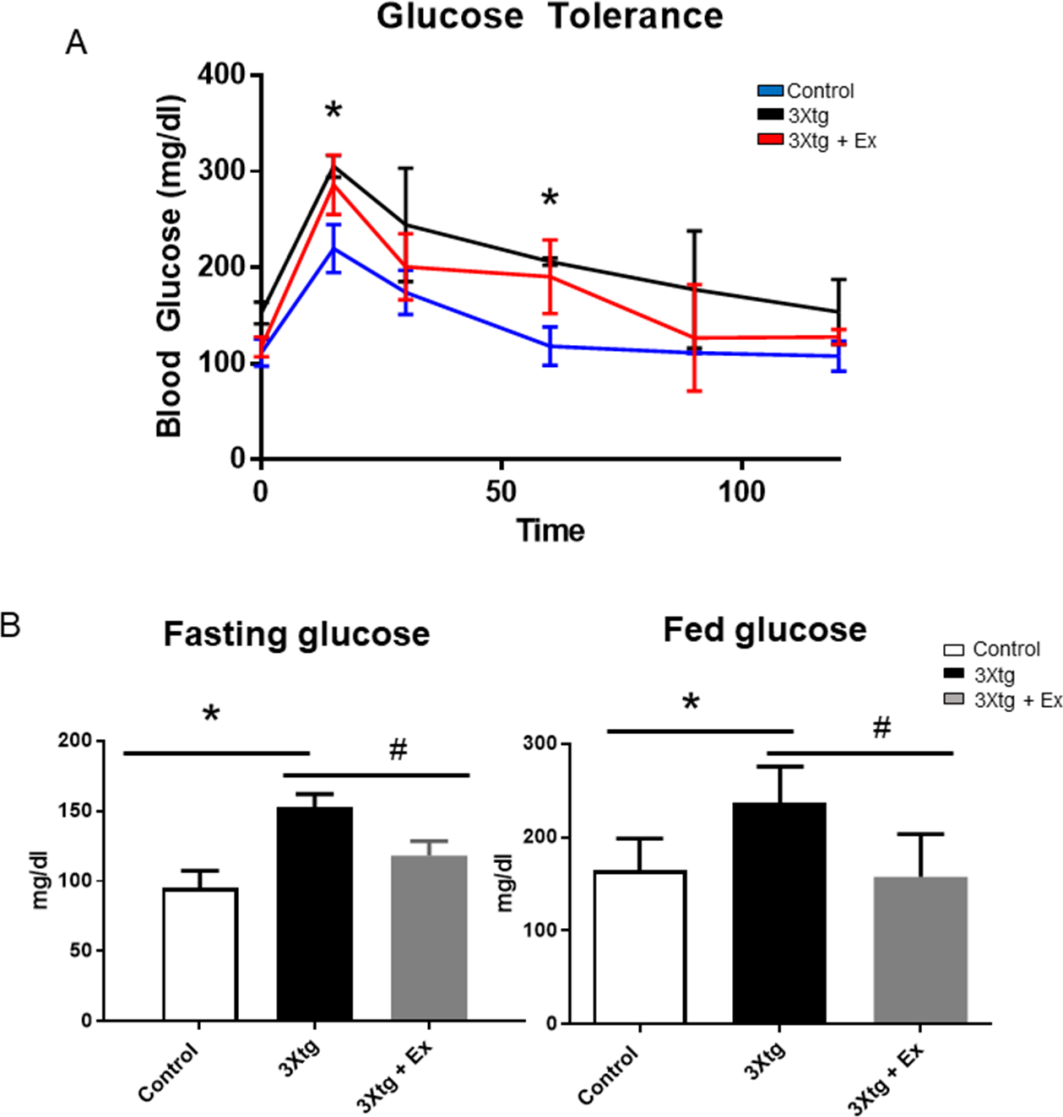
6 weeks’ voluntary exercise training improves glucose metabolism in triple transgenic 3xtg-AD mice. 4A. Fasted and 4B. fed glucose levels at the age of 15 weeks, *p < 0.05 vs control group. 4C. Glucose tolerance test was performed in the control, 3xtg AD, and 3xtg AD + Ex groups at the age of 14 (n = 6), *p < 0.05 vs control group, #p < 0.05 vs 3xtg AD group.

To determine whether long-term voluntary exercise training can improve glucose metabolism impaired by high-fat diet, we first performed glucose tolerance test in the control, 3xtg AD and 3xtg AD + Ex groups after 6 weeks’ exercise training (Fig 4A). There was significant glucose intolerance observed in 3xtg mice compared with control after glucose loaded at 15 minutes (3xtg AD: 306 ± 7 mg/dl versus control: 210 ± 18 mg/dl), and 60 minutes (3xtg AD: 227 ± 2 mg/dl versus control: 118 ± 14 mg/dl) and a 6-week’s exercise training trended to improve glucose tolerance in 3xtg AD mice. After 1 week recovery, both fasted and fed status glucose levels at 15 weeks of age were also measured (Fig 4B). There were significantly elevated fasting plasma glucose levels (3xtg AD: 152 ± 6 mg/dl versus control: 95 ± 8 mg/dl) and fed glucose levels (3xtg AD: 237 ± 16 mg/dl versus control: 164 ± 14 mg/dl) in the 3xtg AD mice compared with the control mice. Interestingly, in 3xtg AD + Ex mice, both fasting glucose levels (3xtg AD + Ex: 118 ± 10 mg/dl versus 3xtg AD: 152 ± 6 mg/dl) and fed plasma glucose levels (3xtg AD + Ex: 158 ± 19 mg/dl versus 3xtg AD: 237 ± 16 mg/dl) were significantly reduced compared to the 3xtg AD mice (Fig 4B). This indicates that 6 week’s exercise training improved glucose metabolism in the 3xtg AD mice (Fig 4).

### POMC-expressing neuron alterations

**Fig 5:**
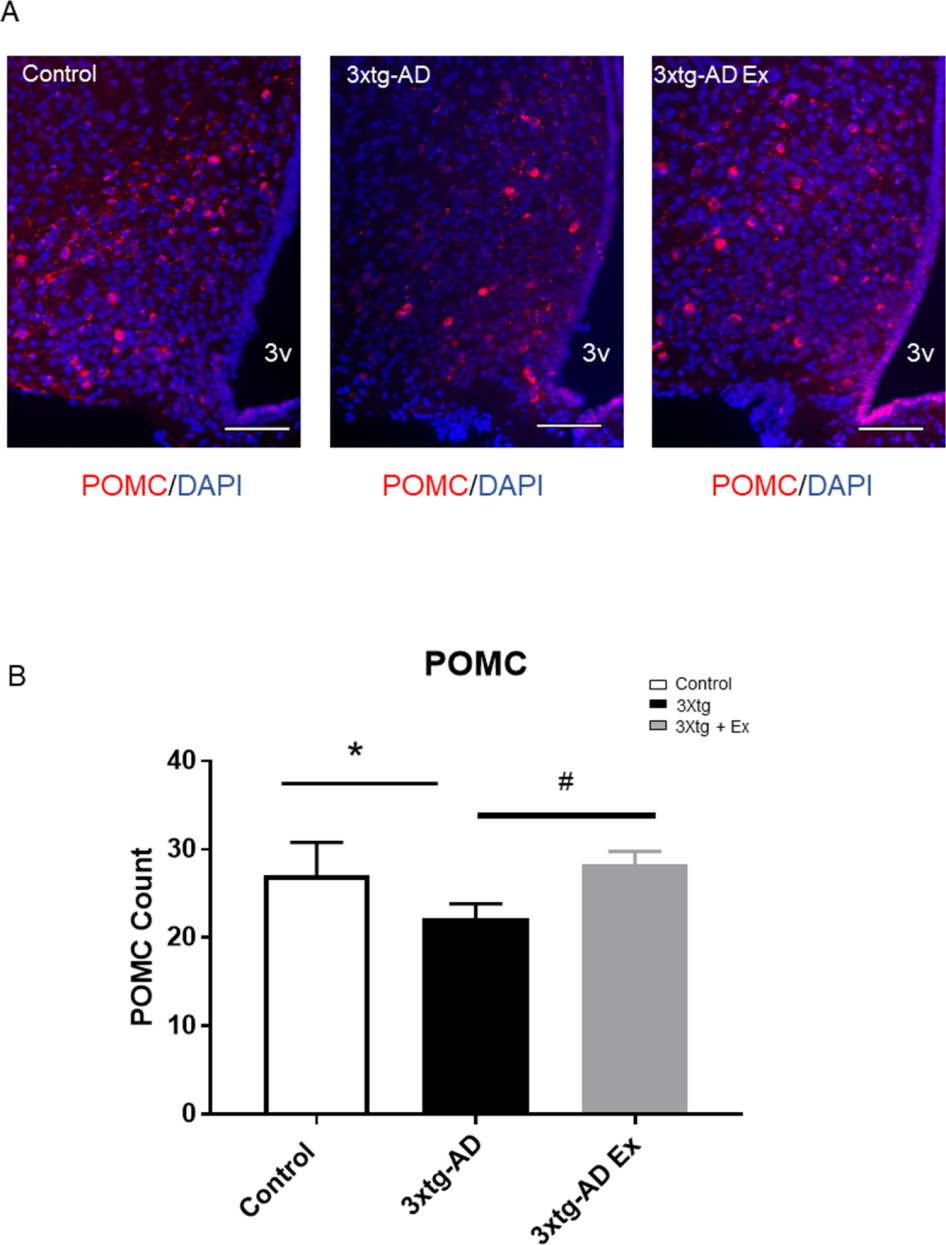
POMC neuron populations after an 8-week voluntary exercise treatment among control, 3xtg-AD, and 3xtg-AD Ex groups. 5A. Representative images of POMC neuron population, 5B. Quantification of POMC neuron populations after 8 weeks of voluntary exercise treatment among control, 3xtg-AD, and 3xtg-AD+Ex group. N = 3–5 per group. *p < 0.05 vs control group, ^#^p < 0.05 vs 3xtg-AD group. 3V: third ventricle, scale bars represent 200 μM, Bregma -1.46mm. Data represent mean ± SEM.

At 12 weeks of age, there was no difference in POMC neuron populations (Data not shown). However, at 24 weeks of age, there was a significant reduction of POMC-expressing neuronal populations in the 3xtg-AD compared to controls (control: 27.1 ± 3.7 counts per section versus 3xtg AD: 22.2 ± 1.6 counts per section (Fig 5A and 5B). 3xtg-AD with 8 weeks of exercise training increased POMC-expressing neuronal population compared to 3xtg-AD (3xtg AD: 22.2 ± 1.6 counts per section versus 3xtg AD+Ex: 28.3 ± 1.5 counts per section) (Fig 5A and 5B).

### NPY-expressing neuron alterations

**Fig 6:**
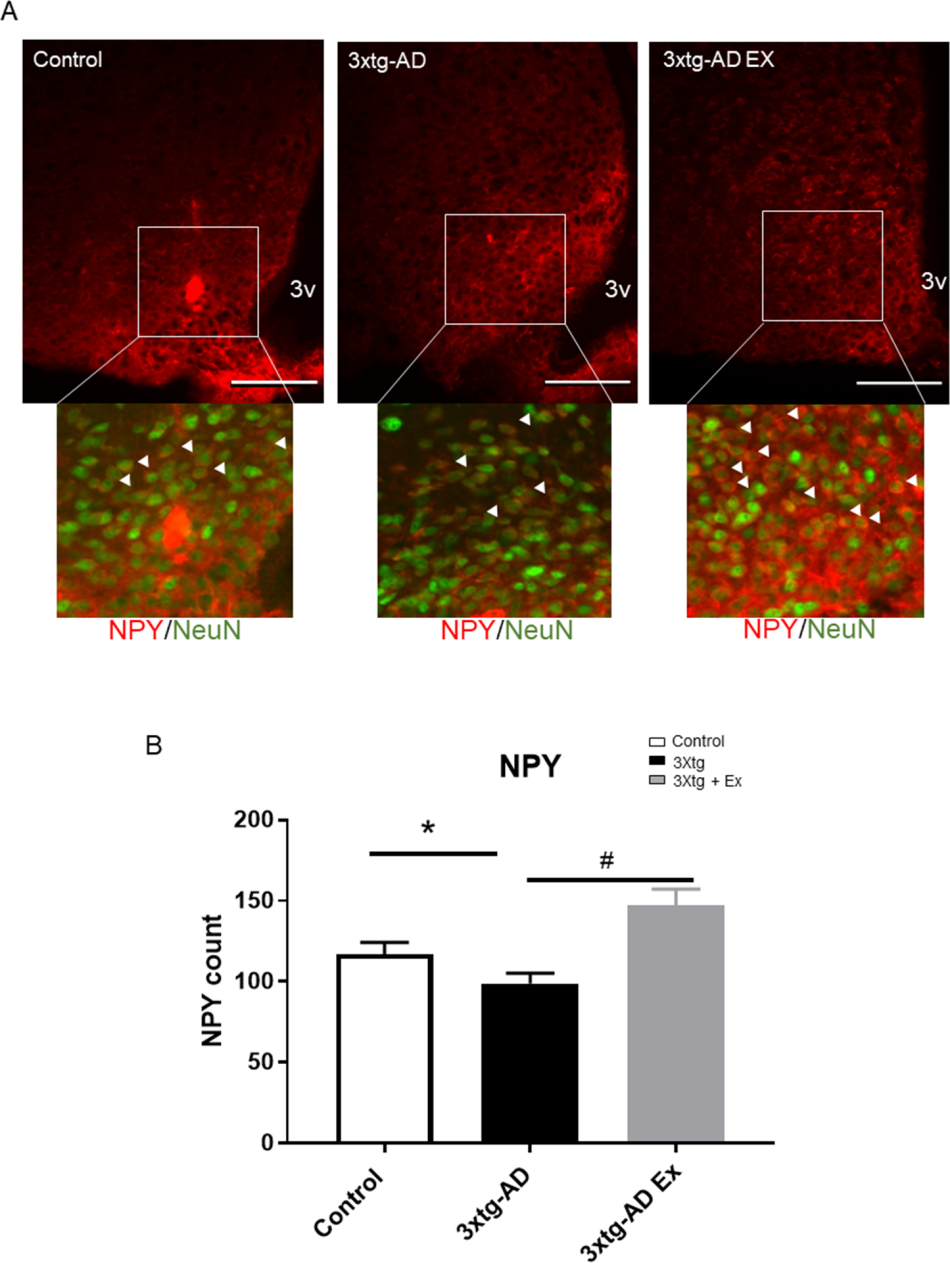
NPY neuron population after 8 weeks of voluntary exercise among control, 3xtg-AD and 3xtg-AD+Ex groups. 6A. Representative images of NPY neuron population, 6B. Quantification of NPY neuron populations after 8 weeks of voluntary exercise treatment among control, 3xtg-AD, and 3xt-AD Ex groups. N = 3–5 per group. White arrows indicate NPY positive cell in NeuN positive neurons. *p < 0.05 vs control group, ^#^p < 0.05 vs 3xtg-AD group. 3V: third ventricle, scale bars represent 200 μM, Bregma -1.46mm. Data represent mean ± SEM.

At 24 weeks of age, there was a significantly decreased NPY-expressing neurons in 3xtg-AD compared to controls (control: 117.1 ± 7.2 counts per section versus 3xtg AD: 98.9 ± 6.4 counts per section) (Fig 6A). In the 3xtg-AD group, 8 weeks of voluntary exercise resulted in significantly higher amounts of NPY neurons than 3xtg-AD group (3xtg AD: 98.9 ± 6.4 counts per section versus 3xtg AD+Ex: 147.2 ± 9.9 counts per section) (Fig 6A and 6B).

### Long-term voluntary exercise training reduces apoptosis in the hypothalamus of 3xtg-AD model

**Fig 7:**
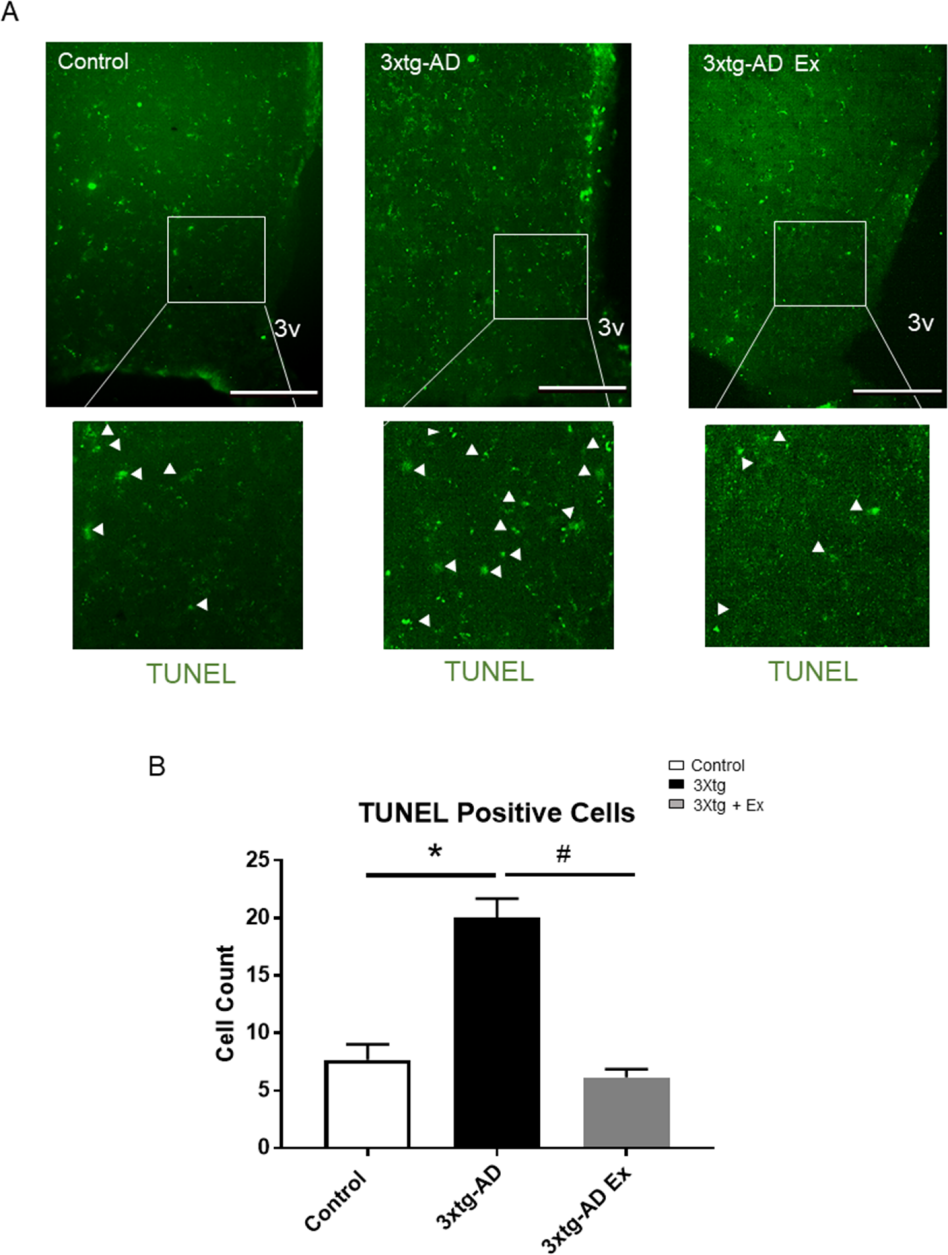
8 weeks’ voluntary exercise training reduces apoptosis in hypothalamus of control, 3xtg-AD and 3xtg-AD+Ex groups. 7A. Immunofluorescence of apoptotic positive cells of hypothalamic sections from the control, 3xtg-AD and 3xtg-AD Ex groups at 16 weeks of age. 7B. Quantification of apoptotic positive cells in the medio-basal hypothalamus among three groups. N = 3–5 per group. White arrows indicate apoptotic cell in the medio-basal hypothalamus. *p < 0.05 vs control group, ^#^p < 0.05 vs 3xtg-AD group. 3V: third ventricle, scale bars represent 200 μM. Data represent mean ± SEM.

To further investigate the potential mechanism of AD and exercise-induced hypothalamic neuron alteration, TUNEL assay was performed to determine the neuronal apoptosis among the three groups (Fig 7). Although there was low level cell apoptosis occurring in the ARC neurons of the control group, 3xtg-AD model mice display significantly augmented cell apoptosis in the hypothalamic neurons, especially in the ARC (control: 7.7 ± 1.4 counts per section versus 3xtg AD: 20.0 ± 1.7 counts per section) (Fig 7A and 7B). 8 weeks’ voluntary exercise training strongly protected the AD-induced apoptosis in this area (3xtg AD: 20.0 ± 1.7 counts per section versus 3xtg AD + Ex: 6.2 ± 0.2 counts per section) (Fig 7A and 7B).

## Discussion

AD is traditionally thought of as a degenerative disease that affects memory and coordination due to its most prominent effects in the cortex and hippocampus. Most studies focus on the cortex and hippocampus because in the late stages of AD progression, amyloid beta plaques build up in the folds of the cortex and induce cognitive impairments associated with the hippocampus [15]. Based on the present study, we have found that AD is not just a neurodegenerative disease; metabolically significant changes occur before the build-up of amyloid plaques and tangles-enriched neurodegeneration, suggesting AD may also be a metabolic disorder. The 3xtg-AD mouse model showed increased energy expenditure through heightened oxygen consumption and increased increase in caloric intake compared with their control counterparts. The absence of increased adipose tissue may be explained by hyper-metabolic rates compensating (overriding) the increased caloric intake. This suggests that there are metabolic abnormalities occurring in the 3xtg AD mouse model at early stages before any hallmarks of AD, such as amyloid plaque formation in the brain [16]. These metabolic abnormalities may play roles in the progression of AD. A comprehensive meta-analysis on the association between obesity and dementia showed that past and ongoing increases in midlife obesity contribute significantly to the future prevalence of dementia [8]. It has also been shown that high-fat/glucose diet in rats induced insulin resistance, impaired spatial learning ability, reduced hippocampal dendritic spine density, and reduced long-term potentiation in the CA1 region [17]. Indeed, not only hippocampus and cortex areas are affected by high-fat diet; a high-fat diet can also rapidly induce neuron injury and eventually cause chronic inflammation in the hypothalamus in obese subjects [18]. In a rodent study, POMC-expressing neurons were specifically and preferentially affected by a high-fat diet in the hypothalamus [19], indicating that metabolic dysfunction often manifests with coexisting neurodegenerative disorders, which may exacerbate neurological symptoms, such as AD. Since hypothalamic neurons regulate many metabolic functions including food intake and energy expenditure [20], we focused on the investigation of hypothalamic neuronal alterations that may be associated with those metabolic abnormalities in the 3xtg-AD mouse model. The gene expression analysis in the hypothalamus of the 3xtg AD mice showed decreased mRNA expression of AgRP and MC4R compared with the control. These decreases in the neuropeptide and its mediators at 12 weeks suggest that these mice may be more susceptible to neuronal damage. A decrease in AgRP neuropeptides, which are orexigenic, should indicate decreased food intake and higher energy expenditure. Interestingly, at 12 weeks, the 3xtg-AD mice display higher food intake despite elevated expenditure, suggesting that there were other neuronal alterations that may have occurred concurrently in the 3xtg AD mice. A select population of neurons in the hypothalamus can alter AgRP/NPY and POMC activation and these neurons are tyrosine hydroxylase (TH)-expressing neurons and release dopamine, which can bind to receptors on the NPY/AgRP and POMC neurons producing an orexigenic effect. This effect is thought to be the origin of pleasure derived from eating, a hedonic effect [21]. It could be that at 12 weeks old, when AgRP neuropeptides are decreased, upregulation of the dopaminergic system in the hypothalamus may act as a compensatory mechanism to help maintain hunger drive in the 3xtg AD mice. At 12 weeks, POMC mRNA showed a decreasing trend. Reduced POMC mRNA may relate to reduced α-MSH, β-endorphin, and ACTH secretions derived from the POMC gene, all of which are neuropeptides responsible for both feeding behaviors and energy expenditure [22]. However, the 3xtg AD mice showed both increased food intake and energy expenditure at 12 weeks; therefore, it is difficult to elucidate exactly which kind of neuropeptide plays a dominant role in the complicated metabolic phenotype of the 3xtg AD mice. Presumably, it is a mixture of multiple neuropeptides and neuronal activation/inactivation involved in this event. To investigate whether reduced neuropeptides were associated with neuron injury or damage in the hypothalamus, we also checked the hypothalamic neuronal populations by stained NeuN, POMC and NPY antibodies as biomarkers for these functional neurons. Strikingly, we observed a remarkable reduction of arcuate neurons (NeuN positive) in the 3xtg AD mice compared with control at 20 weeks of age. Furthermore, immunofluorescence by antibodies to specifically detect POMC and NPY showed that populations of both neurons are decreased in the arcuate area of the hypothalamus. Thus, the irregular metabolic phenotypes in the 3xtg AD mice are highly associated with hypothalamic neuronal alterations at early stages of AD. Although this is before any reported amyloid plaque formation, we cannot rule out the possibility that amyloid oligomers are accountable. Amyloid beta oligomers have been shown to exacerbate inflammation and neurodegeneration through synaptic impairment [16]. Consistent with this finding, amyloid beta has been observed in the cortex of 3xtg AD mice at 12 weeks in the absence of plaque formation. However, there was no detection of amyloid beta in the hypothalamus. Surprisingly, inflammation and apoptosis markers TNF-α and IL-6 were both elevated in the hypothalamus, indicating that early inflammation may be occurring in the hypothalamus of 3Xtg AD animals. It is also in an agreement of TUNEL assay, revealed that apoptosis occurred in the 3xtg AD model of mice.

Exercise interventions have been traditionally administered at older ages and for longer time since cognitive dysfunction is not reported until at least 6 months of age, the starting point of many studies regarding this 3xtg AD model [23]. Additionally, it is well established that exercise decreases the amyloid beta load in the frontal cortex and hippocampus [24]. The effects of exercise in the hypothalamus have not been explored nearly as well. Some studies indicate that exercise training augments excitability in the hypothalamus [25], contributing to augmented peptide signaling, but there have been no reports focused on the effects of exercise on the hypothalamus at the early stages of AD, such as pre-cognitive decline. We further implemented voluntary exercise training, to potentially alter metabolic dysfunction and monitor the differences in the hypothalamic neurons; exercise has been shown to yield many positive benefits related to dementia and metabolic function [26]. In the present study, 4-weeks of voluntary exercise was sufficient to increase the gene expression of AgRP and MC4R in the 3Xtg AD mice. More importantly, the markers for apoptosis and inflammation were completely normalized to the control levels. However, there was no change in the gene expression of PGC1- α, TFAM, and NRF-1, suggesting that short exercise training may not play a significant role in mitochondrial biogenesis in the hypothalamus of 3Xtg AD mice. In this study, we observed that 8-weeks voluntary exercise decreases intracellular amyloid beta in the cortex area, but not in the Dentate Gyrus of hippocampus area in 3xtg-AD mice at age of 5 months [S1 Fig], suggesting that there is differential regulation of brain regional amyloid protein in response to exercise training, it is in line with the original report by Dr. LaFerla et al., shows that extracellular amyloid beta deposits first became apparent in 6 months old in cortex and 12 months in hippocampus [13]. Furthermore, in consistent with the our previous finding [26], we observed a remarkable protective effect on the degeneration of POMC and NPY-expressing neurons in response to 8 weeks of voluntary exercise training via reduced apoptosis of functional hypothalamic neurons in the 3xtg AD model of mice. This suggests that prolonged exercise training could have direct benefits to the hypothalamic neurons in early AD. Taken together, those changes in mRNA expression as well as POMC and NPY-expressing neuronal alterations suggest that exercise training is sufficient to change outcomes related to hypothalamic function in the 3xtg-AD model at a critical point before the development of cognitive decline.

## Conclusion

It is clear that metabolic abnormalities occur pre-cognitive decline in the 3xtg-AD model. Elevated energy expenditure, along with increased energy intake, implies that complicated mechanisms are involved. We observed that, in the pre-pathological stages of AD, there were reductions in neuropeptide gene expressions at 12 weeks, as well as functional POMC and NPY-expressing neurons at 24 weeks. These indicate that at least, hypothalamic-based mechanisms are also associated with the progression of AD, which has not been investigated extensively to date. Additionally, to support the beneficial effects of exercise training on the hippocampus and cortex to prevent AD, we demonstrated that exercise training has a dramatic role on appetite-related neuropeptide, inflammation, and apoptosis gene expressions in the hypothalamus, and may contribute to improved hypothalamic function in the 3xtg-AD mouse model.

## Supporting information

S1 Fig A comparison of amyloid beta in cortex and dentate gyrus area of hippocampus between the sedentary and voluntary exercise in triple transgenic (3xtg-AD) mice. Representative image of amyloid beta in cortex (A) and dentate gyrus area of hippocampus (B) of 3xtg-AD mice in sedentary (top) compared to 3xtg-AD mice in voluntary exercise training (bottom) at 20 weeks of age. scale bars represent 200 μM.
(TIF)

S1 Table Primers information for qPCR analysis. Primer sequences used in qPCR analysis of gene expression.
(RTF)
