# Supplementary figures and images for: The effects of exercise on hypothalamic neurodegeneration of Alzheimer’s disease mouse model
Source: PLoS One. 2018 Jan 2;13(1):e0190205. doi: 10.1371/journal.pone.0190205 (PMC5749759; doi:10.1371/journal.pone.0190205)

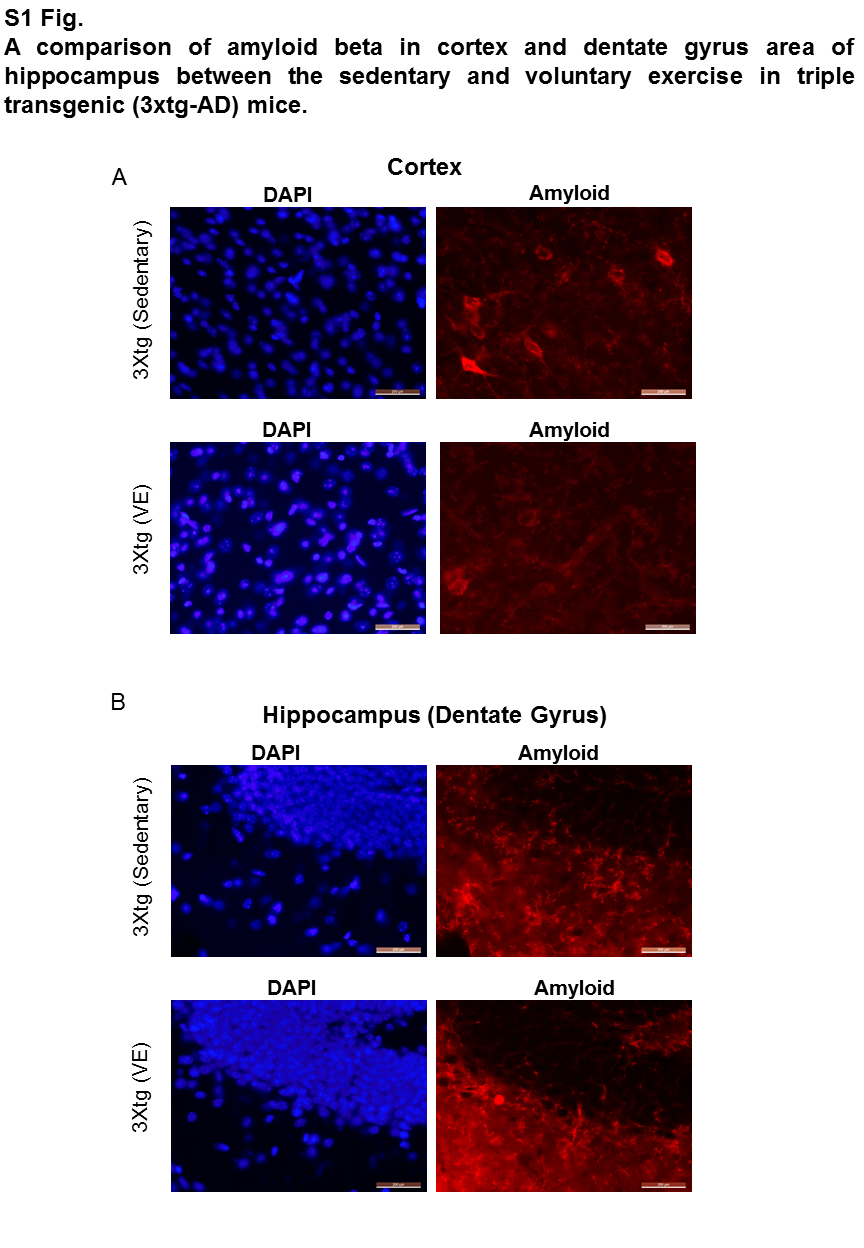

Supplement: S1 Fig — Representative image of amyloid beta in cortex (A) and dentate gyrus area of hippocampus (B) of 3xtg-AD mice in sedentary (top) compared to 3xtg-AD mice in voluntary exercise training (bottom) at 20 weeks of age. scale bars represent 200 μM. (TIF) [file pone.0190205.s001.tif]
