# Supplementary material for: The effects of exercise on hypothalamic neurodegeneration of Alzheimer’s disease mouse model
Source: PLoS One. 2018 Jan 2;13(1):e0190205. doi: 10.1371/journal.pone.0190205 (PMC5749759; doi:10.1371/journal.pone.0190205)
Supplement: S1 Table — Primer sequences used in qPCR analysis of gene expression. (RTF) [file pone.0190205.s002.rtf]

S1 Table: Primers information for qPCR analysis.


POMC:    GCG AGA GGT CGA GTT TGC 
                ACC TCA CCA CGG AGA GCA AC 

AgRP:     GCG GAG GTG CTA GAT CCA CA 
                AGG ACT CGT GCA GCC TTA CAC 

MC4R:    GCG TTT CGA ATG GGT CGG AAA CCA 
                CCG CAA TGG AAA GCA GGC TGC AA 

TNF-á:    CAG GCG GTG CCT ATG TCT C 
                CGA TCA CCC CGA AGT TCA GTA G 

IL-6:        AGT GGT ATA GAC AGG TCT GTT GG 
                CTG CAA GAG ACT TCC ATC CAG 

IKKâ:      CGG CCC TTC CTC CCT AAC 
                GGT GCC ACA TAA GCA TCA GC 

GAPDH:  AAGGTCATCCCAGAGCTGAA  
                CTGCTTCACCACCTTCTTGA                
